# Impact of Fortified Yogurt on Micronutrient Deficits: A Survey on Food Security and Health in the Northeast of Iran

**DOI:** 10.3390/nu16193325

**Published:** 2024-09-30

**Authors:** Mohammad Azadi, Masoomeh Mehraban Sangatash, Ahmad Ehtiati, Hossein Azadi

**Affiliations:** 1 Taleghani Educational, Research and Treatment Center for Accident and Emergency Operations, Mashhad University of Medical Sciences, Mashhad 9189500397, Iran; 2 Department of Food Quality and Safety, Food Science and Technology Research Institute, ACECR Khorasan Razavi Branch, Mashhad 9177949367, Iran; 3 Research and Development Unit, Greeneh Food Industries, Khayam Industrial Zone, Neyshabur 9345114944, Iran; 4 Department of Economics and Rural Development, Gembloux Agro-Bio Tech, University of Liège, 5030 Gembloux, Belgium

**Keywords:** malnutrition, nutritional deficiencies, food quality, consumer acceptance, fortified yogurts

## Abstract

**Background:** Millions of people’s access to food is threatened by the prevalence of micronutrient deficiencies in food, particularly in low- and middle-income nations. **Objectives:** The aim of this study was to evaluate the socio-economic impact of fortified food products on improving the food security of consumers in these regions. **Methods:** This study examined the use of popular products, such as yogurt fortified with inactive baker’s yeast, from April 2023 to December 2023. A questionnaire was developed using a descriptive–inferential approach grounded in practical research. **Results:** The factors of expertise, level of education, and gender significantly influenced the enhancement of food security. Approximately 88% of the variations in food security enhancement factors were attributed to acceptance and consumption, food safety and health, and financial capability and pricing. Among these factors, the acceptance index made the greatest contribution to improving food security. **Conclusions:** Specialized communication and information operations are urgently needed in this area, considering the limited knowledge consumers have about the health effects of newly introduced fortified foods. Therefore, by addressing current shortcomings, this study can help planners, policymakers, and producers of fortified food items increase the demand for fortified goods and improve national food security.

## 1. Introduction

By the middle of the twenty-first century, the world’s population will have surpassed nine billion, raising the demand for food, water, and arable territory and increasing environmental impacts [1]. Food Security concerns, nutritional deficiencies, post-harvest loss rates, and discrepancies in customer behavior and views are all significant challenges that must be addressed in order to maintain food security and sustainability [2,3,4,5]. The World Bank regards food security as an important indicator of development, on par with indicators such as per capita income, employment rate, equitable distribution of income, and respect for human rights, and believes that it must be established as a human investment to achieve a productive and efficient society. The Permanent Food Committee of the United Nations considers food insecurity and hunger the main factors limiting the economic growth and socio-political development of countries and states that having proper nutrition is one of the essential human needs [6]. Every food chain starts with the need for production and is completed with product production, food processing, packaging, distribution, and consumption [7,8]. Increasing the use and effectiveness of solutions such as the production of functional foods in this chain can be influenced by measures such as inter-sectoral coordination, public education, food labeling, monitoring and detailed evaluation, etc. [9,10]. Food Security was described by the World Food Summit as “when all people have physical and fiscal access to sufficient, secure, and nourishing food to meet their dietary requirements and food choices for an active and healthy existence at all times”. The global food security index was created based on these criteria with a particular focus on three characteristics (sub-indices): (a) food availability, (b) financial ability or affordability, and (c) nutritional quality and safety [11,12,13,14,15]. Food processing is any intentional change in food that takes place before its release in the market. As a result, raw food materials become more useful and tastier. Reducing or eliminating the microbial load and toxins, increasing the shelf life, improving the bioavailability of nutrients, and improving the sensory and functional characteristics of food are among the beneficial effects of food processing. Currently, the food industry has a tendency to use natural compounds (compared to chemical compounds) and to remove artificial chemicals, and the use of biologically natural substances in this industry, such as bacteriocins (antimicrobial), ascorbic acid, citric acid (preservatives and nutrients), polyphenols, rosemary extract (antioxidant), types of glucan (functional, nutritious), etc., is expanding [7,16,17].

When utilizing new technology or expertise to find a solution, the issue of “customer approval” is usually one of the most difficult aspects. An excellent concept cannot be economically viable unless it is publicized. To increase success rates, consumer approval and views toward a particular technology must often be addressed early on [18]. It is obvious that it is necessary to know the level of awareness and knowledge of the consumers about the product, and as the first factor of commercial success and reduction in food insecurity, it should be carefully examined [19]. Finally, if effective communication is established between the links of the food chain, the results will be more practical and coherent, and the produced product can be more effective and efficient for improving the food security of society.

Innovation and diversity in products is a type of strategy to achieve competition in the food sector and to fulfill the goals of food industry companies. For this purpose, it is necessary for the managers of these industries to invest and study at the consumer level to predict the results of this innovation. Therefore, before launching and introducing this innovation in the consumer market, a lot of tests and research must be carried out. The success or failure of innovation can be measured from different economic, social, and cultural aspects. In this regard, when it comes to new foods, many food industry owners implement new food technologies. However, essentially, many consumers do not trust this concept (i.e., food technologies). Since the marketing perspective is crucial for the success of a new product, research has recently focused on examining customer behavior [20]. For example, the study results of Goli et al. [6] showed that despite the relative improvement in dimensions such as access, quality and safety, and financial ability of the food security index, the quality and safety of food are still the biggest problems. This is indicative of the nutritional culture of society regarding items such as the variety of food consumed, the absorption of micronutrients, and the quality of absorbed protein [21].

Food security was identified as a global issue for 2050 by Augustin et al. [9]. In their view, the solution to this challenge is not only to improve food production but also to monitor the attitudes and preferences of consumers to know the negative and positive factors affecting the level of consumer awareness. By studying the behavior of consumers of fortified foods, Wahyuni et al. [22] stated that the desire to buy such foods depends on the personal interest of the consumer in relation to the food as well as the appropriateness of the price of the fortified product. Consumers should believe that micronutrient deficiencies are common and that using these types of foods can have personal benefits for them (i.e., improving one’s health level). Bimbo et al. [23] reviewed previous studies on the extent and preferences of functional dairy food products among consumers and found that women have greatly welcomed products such as yogurt enriched with calcium, fiber, and probiotics. Also, the level of acceptance is directly related to the factors of knowledge level and the age of consumers. In addition, the acceptance of products made from natural materials is high. Fear of trying new food is also an effective factor.

Rahnama and Rajabpour [24] noted in their study that there is little knowledge about the role of consumers’ selective behavior in purchasing and consuming dairy products in developing countries like Iran because the majority of studies in Iran focus on the health benefits of consuming dairy products. This factor can cause manufacturers to not be able to identify their customers correctly. Therefore, the main and effective factors in consumers’ behavior toward dairy products are caused by positive feelings (enjoyment, comfort, and relaxation) [24]. A study on the effects of the increase in the price of food products on the welfare and poverty of urban households showed that, as a result of the increase in the initial prices of enriched foods, the number of poor families and those below the poverty line is increasing [25].

The food industry in Iran is responsible for important matters such as ensuring food security, creating employment, and ensuring people’s health. Considering the perishability of most food products, cost reduction, waste reduction, and compatibility with environmental standards are important issues, and establishing a lean supply chain and security in production can be effective in the sustainability of these industries and, as a result, the health of society. The food products that are produced with the purpose of improving specific physiological processes are known as functional foods in many food groups. Enriched with specific compounds, some of them enhance physiological functions and also reduce the risk of disease. As a result, these foods are marketed as foods that have the ability to improve the well-being of consumers. Despite the importance of these types of foods for physical and mental health, their use is not very common at present. The type of behavior and choice of food by consumers depends on many factors, such as awareness and demographic factors such as gender, age, and education. In addition, attitude and lifestyle factors strongly influence consumer behavior [25]. Therefore, there is a clear need in the food and nutrition sectors for additional studies on how consumers make food choices.

According to a report by the Ministry of Cooperatives, Labor, and Social Welfare [26], dairy products, including yogurt, are one of the main components in food baskets distributed among Iranian households, and considering its price range, it is also among the most consumed products. Due to these features, yogurt can be used as the selected product for enrichment, with the aim of reaching all sections of society. So far, various studies have been conducted on the enrichment of yogurt [27,28]. However, a study that examines the socio-economic effects of accepting and consuming fortified food products on improving the food security of society has not yet been performed. This is the most important innovation of this study, which tries to answer the following questions by filling the gap in past studies:

1. Does the food quality and safety (food health) of yogurt fortified with inactive baker’s yeast improve food quality?
2. Is the acceptance (consumption) of yogurt fortified with inactive baker’s yeast effective in improving food security?
3. Is the price of yogurt fortified with inactive baker’s yeast effective in improving food security?

The current study will help food industry owners, policymakers, and socio-economic planners know the acceptance and sales of a new food product before final production and how it affects food security and community health.

## 2. Materials and Methods

### 2.1. Type and Method of Research

This study investigates the role of using yogurt enriched with inactive baker’s yeast in enhancing food security, focusing on three key aspects: food quality and safety, consumer acceptance, and price. The study was conducted during the period from April 2023 to December 2023 using a descriptive–inferential applied research design. This study investigated the link between the independent and dependent variables, the significance of variations in the independent variable means, and the ability to predict changes in the dependent variable as a result of changes in the independent variables.

The participants in this study were chosen by a random selection process. This study’s statistical population includes professionals and specialists in nutrition research, economics, agricultural development, and the food business. The expert status of participants was defined based on their educational qualifications and professional experience. Specifically, participants were considered experts if they had an advanced degree (Master’s or Ph.D.) in their respective field or significant work experience of at least five years in their respective field. The questionnaire used in the research was prepared based on theoretical foundations and previous research related to the research objectives. To ensure its content validity, a thorough validation process was carried out, including the opinions of professors and experts in the relevant fields. In addition, the questionnaire was pre-tested with a small group of non-academic experts, which led to modifications before it was finalized for use in the study. Cronbach’s alpha was used to confirm the questionnaire’s reliability, and it was determined that it is an effective data gathering method.

Due to the large size of the statistical population in this research, the random sampling method was used. When the studied community is formed by a small group in a certain area, statistically, that community is considered a limited population, and therefore, it is better to use Cochran’s formula, which is more accurate compared to other formulas such as Morgan’s, to determine the sample size. Determining the variance is essential to calculate Cochran’s formula. Therefore, 30 questionnaires were completed by the preliminary sample (non-university experts who were not included in the main sample), and the size of the research sample was estimated through the following equation:

(1) $$n=\frac{Nt2S2}{\left(Nd2+t2S2\right)}=211$$

(2) $$d=t\frac{s}{n}$$

where

*n* = the acceptable sample size.
*N* = the size of the whole population.
*t* = the fixed value of “*t*” with 95% confidence or 5% error (*t* = 1.96).
*S* = the variance in the dependent variable in the preliminary test (S2).
*d* = the error allowed.

Finally, the statistical population under study was estimated to be 211 people.

### 2.2. Data Collection and Statistical Analysis

In this study, the necessary information was collected in two parts. The first part included the collection of information on the theoretical foundations of the subject, as well as reviewing previous research, by conducting a literature review using library and internet resources between April and June 2023. The second part included the collection of the required information from both academic and non-academic experts under study. This was achieved using a questionnaire designed based on the theoretical foundations, objectives, and hypotheses of the research and was conducted in June 2023. In this questionnaire, in addition to evaluating demographic characteristics (individual, educational, etc.), between 10 and 15 questions were designed for each sub-index. This questionnaire was designed with four sub-indices as follows: (a) individual characteristics; (b) food quality and safety (food health); (c) the rate of acceptance (consumption availability of food); and (d) financial ability (price affordability).

The Likert scale was used to measure the range of responses related to opinions sought in the questions of the questionnaire, in which five opinions were considered (very low, low, medium, high, and very high). In order to determine the validity of the questionnaire, the opinions and suggestions of professors of rural development, education promotion groups, agricultural economics, food science and technologies, and nutrition science were used, and after making the necessary corrections and ensuring that the questions raised had the ability to measure the contents and features desired in the research, the research process continued. Cronbach’s alpha index was also employed to assess scientific validity or reliability, and because it was more than 0.76 for all the study questions, the questionnaire’s validity was affirmed. The questionnaires were completed both face-to-face and online between July and September 2023. Finally, statistical analysis of the collected data was conducted using descriptive and analytical statistical methods via SPSS_23_ software from October to December 2023.

## 3. Results

### 3.1. Descriptive Statistics (Personal Characteristics)

In descriptive statistics, the rate of repetition of phenomena, central tendency, variability, and dispersion are investigated. Descriptive statistics simply give an overview of the population under study; they cannot identify or address the dependent variable or variables. In this study, 211 specialists and experts fully answered the questions in the final questionnaire (Cronbach’s alpha equal to 87%). To describe the independent and dependent variables in the research, statistical characteristics such as frequency distribution tables, the frequency percentage, cumulative frequency, and minimum and maximum were used.

Descriptive statistics reveal that among the examined participants (N = 211), 109 of them were women with the highest frequency (F = 109) and 102 were men (F = 102). As a result, most of the people examined in this study are women. Also, among the people studied in this research, 37.9% of them with a frequency of F = 80 were in the age group of 31 to 40 years. After that, the highest frequencies were related to the age groups of 41 to 50 years (F = 66), 21 to 30 years (F = 37), 51 to 60 years (F = 24), and above 60 years (F = 4). As a result, most of the studied people are in the middle age group.

The results show that, in terms of education level, about 46% of the studied people with the highest frequency (F = 97) had a Ph.D. degree, while 32.7% of them had a Master’s degree and 20.4% had a bachelor’s degree, and only 0.9% of them had other degrees. Therefore, most of the surveyed people had a Ph.D. degree.

According to the results, 26.1% of the studied people were from the nutrition sciences group; 21.3% were from the food technologies group; 18% were from the extension, education, and agricultural economy group; and 34.6% of them, with the highest frequency (F = 73), were active in other fields. Examining the work experiences of the people under study shows that 27.5% of the people with a frequency of F = 58 had less than five years of work experience, while 26.1% of them had between 11 and 20 years, 21.8% had between 21 and 30 years, 20.9% had between 5 and 10 years, and about 3.8% had more than 30 years of work experience.

### 3.2. Inferential Statistics

The Kruskal–Wallis test was performed to assess the association between the tested participants’ knowledge and improvements in food security. The results show that the obtained value of sig = 0.002 is significant at the 95% confidence level (see Table 1). Accordingly, it can be stated that there is a significant difference between the expertise of the people studied and the improvement in food security. Therefore, according to the knowledge and awareness of these people regarding the benefits of functional foods, they can play an important role in accepting these types of products at the community level.

Furthermore, the findings indicate that there is a significant difference (sig = 0.009) between the education level of the tested participants and the increase in food security. In other words, the higher people’s levels of knowledge, the more their acceptability, and hence the larger their impact on enhancing food security will be.

As demonstrated in Table 2, calculating the correlation coefficient between the two variables, age and improving food security (sig = 0.004 and r = −0.195), indicates that there is a negative but significant relationship between the two variables at the level of 1% error. In other words, when people are younger, the improvement in food security is greater. The results (Table 2) also indicated the correlation coefficient between the two variables job experience and improved food security (r = 0.109 and sig = 0.193). They indicate that there is no significant relationship between the two mentioned variables. Therefore, work experience does not contribute to improving food security.

A comparison of means (T) was used to examine the impact of gender on the dependent variable of increasing food security (comparison between the two groups of men and women). The results in Table 3 show that the variance in the two variables (sig = 0.015) is almost the same. This suggests that gender has a substantial impact on enhancing food security. According to the t values, it can be said that women have a greater effect on improving food security.

According to the results obtained from the Pearson correlation matrix table (Table 4), with 99% confidence and an error level of less than 0.01, the dependent variable of improving food security (M.V) has a significant and positive relationship with the independent variables of food quality and safety (Q), consumer acceptance (C), and product price (A). In addition, the value of this relationship, which is equal to 0.776, 0.822, and 0.733, respectively, is direct and strong. In this regard, hypothesis H1 is rejected, and hypothesis H0, which is the hypothesis of this research, is accepted.

### 3.3. The Results of Stepwise Regression

Regression analysis is a popular technique in socio-economic research. This technique is closely linked to the correlation coefficient and is commonly used at the same time in studies. Regression analysis enables researchers to predict changes in the dependent variable using independent factors and to determine the input of each independent variable in order to better understand the dependent variable. The stepwise technique is a multivariate regression method that adds the variable with the highest effect into the equation after analyzing all of the independent variables. This process is repeated until no variable is able to enter the multivariate regression equation. In this study, three variables were incorporated into the multivariable regression equation using the stepwise technique, as shown below.

Step One: In this step, the first variable added to the equation is x1, which represents the quantity of acceptance (consumption). This indicates that this variable has the largest influence. At this point, the correlation coefficient (R = 0.822), coefficient of determination (R Square = 0.676), and adjusted coefficient of determination (Adjusted R Square = 0.675) are computed. On the other hand, the F value obtained from the variance analysis is 436.816, and its significance level is sig = 0.000, which is significant at a level less than one thousandth. Based on the coefficient of determination, it is possible to conclude that the independent variable C, i.e., the acceptance rate, accounts for approximately 67% of changes in the dependent variable of improved food security. As a result, the equation of the regression line Y = a + b1 × 1 + b2 × 2 + … in the first step is Y = 1.889 × 1 + 42.959, and its standardized equation is equal to Y = 0.822 × 1.

Step Two: Following the x1 variable, the x2 variable, which represents food quality and safety (food health), is introduced into the equation. At this point, the correlation coefficient is R = 0.908, the determination coefficient is R Square = 0.824, and the corrected determination coefficient is R Square = 0.822. Furthermore, the F value from the analysis of variance is 486.841, indicating significance at the one-thousandth error level. The coefficient of determination found indicates that the variables of acceptance rate, food quality, and safety account for about 82% of the variations in the dependent variable. As a consequence, the regression line’s equation in the second step is Y = 1.301 × 1 + 1.213 × 2 + 11.309, with its standardized equation being Y = 0.566 × 1 + 0.462 × 2.

Step Three: At this stage, after entering the acceptance rate and quality and Food Security variables, the price variable (financial ability), i.e., ×3, is entered into the multivariable regression equation. The correlation coefficient is equal to R = 0.941, the determination coefficient is equal to R Square = 0.886, and the adjusted determination coefficient is equal to Adjusted R Square = 0.884. Also, the F value obtained is equal to 536.936, which is significant at the level of less than one thousandth. By observing the obtained coefficient of determination, it can be stated that about 88% of changes in the dependent variable of improving food security are caused by the variables ×1, ×2, and ×3, i.e., acceptance and consumption, food quality and safety, and price and financial ability, respectively. As a result, the equation of the regression line in the third step (the last step) is Y = 1.006 × 1 + 1.016 × 2 + 0.934 × 3 + 3.539, and its standardized equation is equal to Y = 0.438 × 1 + 0.387 × 2 + 0.308 × 3.

According to the results of the stepwise regression, it can be stated that among the three independent variables investigated, variable C, or the variable of acceptance (consumption) with the highest standardized regression coefficient (β = 0.438), has the highest contribution to improving food security (Table 5). Since the standardized regression coefficient for all three variables is significant at an error level of less than 0.01, it can be said that variables such as consumption, food health, and financial ability are effective in improving food security. As a result, the research hypotheses regarding the positive and significant effect of independent variables (food quality and safety, acceptance rate, and financial ability) on the dependent variable (improving food security) are confirmed.

## 4. Discussion

Based on the findings of this study, demographic factors (i.e., gender, age, and education level) significantly affect the improvement of food security. The findings indicate that women play a major role in increasing food security, which might be attributed to them frequently preparing household meals and their increased interest in healthy eating among family members, particularly their children. Compared to men, women demonstrate greater attention to the nutritional content of products while shopping. Women are generally the primary purchasers and consumers of functional foods, likely due to their greater interest in healthy eating. This heightened interest is particularly important given their key role in managing food purchases for the household. According to these findings, it seems that in planning to promote the consumption of functional dairy products in society, it is better to focus more on women, as they are the potential consumers of these products. This finding was confirmed by Bazhan et al. [29]. Although some researchers say that one of the important pillars of food security is achieving security or health, i.e., protein, energy, micronutrients, and substances that provide health for all family members [30], ensuring the household’s food security through the combination of food and other resources is important for women. According to Browne et al. [30], one necessity for battling hunger and poverty and attaining food security, particularly at the household level, is to support women’s empowerment. They also emphasized the importance of women’s role in ensuring family food security.

The results also indicate that increasing people’s knowledge and awareness of the benefits of functional foods can play an important role in facilitating the acceptance of these types of products at the community level.

As shown by the findings of this study, education is one of the important indicators in the socio-economic status of any society and is known as one of the factors that facilitates healthy eating and food security. Many studies, including those by Annunziata and Vecchio [31], Bholah and Neergheen-Bhujun [32], and Brečić et al. [33], found that functional food consumers have a greater degree of education than those who do not use these goods. As a result, education is an essential component in the adoption of these kinds of goods, which can play a significant role in enhancing societal food security.

According to Shen et al. [34], the role of food in human health and nutrition is very important in the sense that the greatest importance of food, rather than its primary role as a source of energy and growth, is its biological role in human health, and the food production and consumption market has shifted more toward the production of functional foods. The increase in participation in education at the community level has increased the awareness of consumers, and day by day, this creates new developments in the production of such foods, which can guarantee food security. In other studies, such as those conducted by Calfee and Piontkowski [35] and Bazhan et al. [29], a statistically significant connection was found between education and the intake of functional dairy products. They demonstrated that as the level of education rises, so will the approval and usage of functional dairy products in society, confirming this study’s results.

Ahmad et al. [36] state that the acceptance of enriched yogurt consumption among young and middle-aged people was much higher compared to older age groups. Due to the increase in awareness and knowledge, these people have a better understanding of food security, and for this reason, improving the quality of nutrition through the consumption of nutritious food is more important for them. The findings of this study also confirm this issue.

According to the results, raising the level of quality and safety of the product and its level of food safety will increase food security significantly. Also, increasing the level of acceptance and consumption of enriched yogurt by consumers will, to a large extent, improve the level of food security. In addition, the increase in the price of enriched yogurt will not have a negative effect on its consumption, and food security will be significantly improved among its consumers. These results show that the acceptance of enriched yogurt as a valuable product with high nutritional health in household food baskets, in spite of its high price, can improve food security in society.

While the issue of food security and safety is still an unsolved problem in developing countries, the production and consumption of these food products in developed countries have grown significantly. As for the minimum benefits of producing and consuming a variety of useful food products such as enriched yogurts in every society, we may refer to increasing the general health of society and improving food security, paying more attention to prevention instead of treatment, reducing the difficulties and costs of treatment, especially in the case of children and the elderly, creating added value to raw materials after their sale, creating competition and boosting the food economy, contributing to innovation and scientific creativity, and increasing human dignity and rank due to valuing health and food safety. In line with these findings, Coetzee [37] shows that it is possible to improve the nutritional consumption of households by enriching bread with flour. According to her findings, the nutritional value and acceptable sensory characteristics of enriched bread can improve the food security status of households. In addition to this, the findings of De Groote et al. [38] show that corn flour consumers prefer sorghum-enriched flour to regular flour. She states that although fortified flours are nutritionally complete, they are not cost-effective. Nevertheless, these types of products are still welcomed by consumers.

The success of fortified food products, especially functional foods, depends on their acceptance and consumption by target populations. Consumer acceptance is measured in terms of comparing the sensory evaluation and economic valuation of these types of products with those of their normal types. Different factors can affect consumers’ evaluation and valuation of fortified foods. These factors are as follows: 1—nutrition information and the media through which such information is transmitted; 2—different branding options; 3—the price of these products; and 4—ensuring the nutritional health of these types of products. On the other hand, the efficiency and cost-effectiveness of fortified products are suitable strategies to reduce micronutrient deficiencies. Therefore, information is needed on how fortified products are accepted by consumers and what kind of mechanisms or factors (as a pull mechanism for acceptance) can maximize the consumption of fortified products.

Some of the reasons for the human need for enriched food include the increase in the elderly population, people’s desire to improve their quality of life, improving food security with the aim of optimizing nutrition and enhancing body immunity in stress conditions, reducing the risk of disease, and prevention and treatment. The development and commercialization of fortified products are very complex and expensive because the specific needs of the consumer must be addressed. This development requires effort and research. According to Bazhan et al. [29], the market for fortified (especially functional) foods is expanding globally and seeing a continuous influx of new goods. She believes that the expansion of these products requires the awareness of the factors affecting their acceptance and states that the facilitators of consuming functional dairy can be explained in the form of five main categories: (1) product-related factors (sensory and no sensory characteristics); (2) price-related factors (physical access and economic access); (3) location; (4) promotion-related factors (information, education, expert or peer recommendation, advertising, and product support as social marketing ingredients); and (5) consumer-related factors as a new dimension (adequate knowledge, socio-demographic characteristics, favorable attitudes toward the product, food taste, values, and food pricing) [29].

There is a critical need for specialized communication and information efforts in this area given the consumers’ limited understanding and knowledge of the health impacts of functional compounds that have recently joined the market. As a general conclusion, it is important to highlight that information about functional dairy at the community level is insufficient, contradictory, and confusing. Therefore, the majority of people want to know more about functional dairy products through more advertising of these products on TV, introducing these products in scientific programs on the country’s broadcasting system, and including more and more information on the product label. The existence of sufficient documents and evidence based on the effectiveness of functional dairy products and the advice of health professionals (doctors or nutritionists) are the most important recommended conditions for the acceptance of functional dairy products. Government institutions and organizations, nutritionists, and doctors are the most reliable sources of information, and sellers, friends and acquaintances, and producers are the most effective sources promoting this product. Also, promoting the use of these products requires appropriate pricing, similarity with the prices of functional dairy products and non-functional dairy products, allocation of subsidies, easy access and production, and the supply of more functional products.

This study highlights key policy implications for improving food security by promoting functional foods in household food baskets. For this purpose, policymakers should develop educational campaigns and public awareness programs that highlight the benefits of functional foods. It is also necessary to integrate nutrition education into public health plans and use media platforms to reach diverse demographic groups. Considering the important role of women in household food security and the importance of targeting young and old populations, appropriate support programs by the government (for example, considering food subsidies) should be implemented to increase access to functional foods for these vulnerable groups. In addition, it is very important to strengthen regulatory standards for more precise control of food quality and safety because increased regulatory efforts will greatly ensure that functional foods comply with high food and safety standards. To make functional foods more affordable and accessible, policymakers should consider implementing subsidies and financial incentives for underserved areas and encourage public–private partnerships to drive innovation and distribution. Finally, establishing robust monitoring and evaluation frameworks will enable data-driven adjustments in policies and ensure their effectiveness in improving food security and public health outcomes.

### Research Limitations

Due to the fact that this study was conducted during the COVID-19 pandemic and because of people’s fear of this pandemic, a smaller number of selected people declared their readiness to participate in the survey. Also, due to the quarantine conditions governing society, access to many statistics and information was limited, especially those from companies producing enriched yogurt.

Given that this study examined the socio-economic effects of using enriched products to improve food security, future research should explore the behavioral and social norms related to the acceptance of these products at the micro level. It is also recommended to identify the key factors contributing to the non-acceptance of these products in less developed societies through qualitative analysis and the development of a paradigm model. Therefore, by using this model, politicians, planners, and workers in manufacturing industries can remove obstacles and turn these products into a safe alternative to improve the health of society.

## 5. Conclusions

Globally and in some countries, there are continuing arguments concerning the effectiveness and safety of enrichment initiatives (whether eating foods enriched with micronutrients may have negative health consequences owing to the buildup of these nutrients in the human body). The results of this study emphasize that yogurt fortified with inactive baker’s yeast could improve food security. Therefore, this requires a change in the consumer’s attitude and an increase in the acceptance (consumption) of food enriched with yeast, which will ultimately lead to an increase in the quality of effective food security in society. In this regard, the price of enriched products is influential in increasing the acceptance of the product, although educated people who have a positive attitude towards the use of these types of products and include them in their family’s food basket agree that price is not an important factor in ensuring health and increasing food security. Food fortification is a low-cost technique for improving population nutrition that will yield significant economic advantages.

Everyone should have access to healthy and nourishing food, as mentioned in the World Food Summit Declaration. As a result, fighting starvation and malnutrition is more than a moral responsibility in many countries; it is also a constitutionally enforceable component of national laws under human rights responsibilities. Unfortunately, in many countries, ensuring the right to food and nourishment is a difficult and sometimes overwhelming responsibility, especially in the context of the COVID-19 pandemic, where mandated quarantines and decreased economic development contributed to increased food insecurity and malnutrition. Food fortification, as a result, can be an essential measure to decrease the risk of malnutrition before, during, and after a crisis.

Food fortification is a critical component in fostering public–private collaborations that benefit society across multiple sectors. While private-sector food company partners are the key participants in food fortification projects, governments and civil society also play important roles. Civil society, in particular, may help to improve transparency and commitment standards, among other things. Public–private partnerships are critical since the majority of food fortification efforts include both public–private coalitions and customer contacts. When combined with social safety net programs such as social feeding, distribution to poor and vulnerable groups, food for work programs, and food aid in emergency situations, civil society, donors, and food product enrichment will be effective tools for providing fortified food to vulnerable people and disseminating diet information. Global and national experiences show that food fortification is most likely to be achieved through collaboration not only between the public and private sectors, but also between parties and organizations capable of working in critical areas (support, management, capacity building, implementation, and supervision).

In addition to these elements, raising literacy and community awareness, focusing on women as those who prepare and plan the majority of household meals, taking product expenses into consideration, designing field studies, and engaging in interdisciplinary investigations into the subject of social and cultural food security, including sectors like promotion, education, agricultural economics, food technologies, and nutrition sciences, will all have an impact. Also, the food industry sector can play an important role in improving the food security of society, considering the priority of food needs of society, the targeted production of functional foods, and their introduction to society. In fact, the attention of the food industry to the health and food security of society, predicting the level of acceptance of a manufactured product at the community level by using field studies and market research, paying attention to food chains and the factors affecting these chains, and also receiving the opinions of specialists and experts can yield highly valuable results. Therefore, in order to improve the food security of society, it is better to enrich the foods that are accepted by the people of society and, in terms of food culture and also price, those that are consumed by the majority of the people in society, especially low-income people. Accordingly, yogurt, which is a dairy product, can be used for this purpose.

## Figures and Tables

**Table 1:** The results of the Kruskal–Wallis test.

Row	Independent Variable	Dependent Variable	sig	Chi-Square	DF = k − 1
1	Expertise	Improving food security	0.002	11.649	4
2	Education	Improving food security	0.009	13.485	4

**Table 2:** The results of Spearman’s correlation coefficient.

Row	Independent Variable	Dependent Variable	Value of r	sig
1	Age	Improving food security	r = −0.195	0.004 **
2	Work experience	Improving food security	r = 0.109	0.193

** Significance at the level of 1% error.

**Table 3:** The results of the *t*-test.

Group	Number	Mean	T	df	sig
Women	109	82.133	79.01	209	0.015
Men	102	32.129	773.1	187	

**Table 4:** The results of Pearson’s correlation coefficient.

	Improving Food Security	Food Quality and Safety	Acceptance	Price	Improving Food Security
Improving Food Security	Pearson Correlation	1	0.776 **	0.822 **	0.733 **
	Sig. (2-tailed)		0	0	0
	N	211	211	211	211
Food Quality and Safety	Pearson Correlation	0.776 **		0.555 **	0.474 **
	Sig. (2-tailed)	0		0	0
	N	211	211	211	211
Acceptance and Consumption	Pearson Correlation	0.822 **	0.555 **		0.552 **
	Sig. (2-tailed)	0	0		0
	N	211	2111	211	211
Price	Pearson Correlation	0.733 **	0.474 **	0.552 **	1
	Sig. (2-tailed)	0	0	0	
	N	211	211	211	211

** Correlation is significant at the 0.01 level (2-tailed). Dependent variable (improvement in food security): M.V. Food quality and safety index (food health: 14 questions): Q = Q1–Q13. Acceptance index (consumption: 15 questions): C = C1–C15. Price index (financial ability: 10 questions): A = A1–A10.

**Table 5:** The coefficients of the variables entered in the regression equation.

Model		Unstandardized Coefficients		Standardized Coefficients	t	Sig.
		B	Std. Error	Beta		
1	(Constant)	959.42	386.4		795.9	0
	(C) Acceptance and consumption	889.1	90	822	9.2	0
2	(Constant)	309.11	32.4		805.2	6
	(C) Acceptance and consumption	301.1	80	566	202.16	0
	(Q) Food quality and safety	213.1	92	462	207.13	0
3	(Constant)	539.3	332.3		62.1	289
	(C) Acceptance and consumption	539.3	70	438	287.14	0
	(Q) Food quality and safety	16.1	76	387	311.13	0
	(A) Price	934	88	308	629.1	0

Dependent variable: M.V.

## Data Availability

The original contributions presented in the study are included in the article, further inquiries can be directed to the corresponding author.
